# High-throughput assays to identify archaea-targeting nitrification inhibitors

**DOI:** 10.3389/fpls.2023.1283047

**Published:** 2024-01-08

**Authors:** Fabian Beeckman, Andrzej Drozdzecki, Alexa De Knijf, Dominique Audenaert, Tom Beeckman, Hans Motte

**Affiliations:** ^1^ Department of Plant Biotechnology and Bioinformatics, Ghent University, Ghent, Belgium; ^2^ Center for Plant Systems Biology, Vlaams Instituut voor Biotechnologie (VIB), Ghent, Belgium; ^3^ Screening Core, Vlaams Instituut voor Biotechnologie (VIB), Ghent, Belgium; ^4^ Centre for Bioassay Development and Screening (C-BIOS), Ghent University, Ghent, Belgium

**Keywords:** nitrification inhibitors, *Nitrososphaera viennensis*, high-throughput (HT) screening, ammonia-oxidizing archaea, nitrifying community

## Abstract

Nitrification is a microbial process that converts ammonia (NH_3_) to nitrite (NO_2_^-^) and then to nitrate (NO_3_^-^). The first and rate-limiting step in nitrification is ammonia oxidation, which is conducted by both bacteria and archaea. In agriculture, it is important to control this process as high nitrification rates result in NO_3_^-^ leaching, reduced nitrogen (N) availability for the plants and environmental problems such as eutrophication and greenhouse gas emissions. Nitrification inhibitors can be used to block nitrification, and as such reduce N pollution and improve fertilizer use efficiency (FUE) in agriculture. Currently applied inhibitors target the bacteria, and do not block nitrification by ammonia-oxidizing archaea (AOA). While it was long believed that nitrification in agroecosystems was primarily driven by bacteria, recent research has unveiled potential significant contributions from ammonia-oxidizing archaea (AOA), especially when bacterial activity is inhibited. Hence, there is also a need for AOA-targeting nitrification inhibitors. However, to date, almost no AOA-targeting inhibitors are described. Furthermore, AOA are difficult to handle, hindering their use to test or identify possible AOA-targeting nitrification inhibitors. To address the need for AOA-targeting nitrification inhibitors, we developed two miniaturized nitrification inhibition assays using an AOA-enriched nitrifying community or the AOA *Nitrosospaera viennensis*. These assays enable high-throughput testing of candidate AOA inhibitors. We here present detailed guidelines on the protocols and illustrate their use with some examples. We believe that these assays can contribute to the discovery of future AOA-targeting nitrification inhibitors, which could complement the currently applied inhibitors to increase nitrification inhibition efficiency in the field and as such contribute to a more sustainable agriculture.

## 1 Introduction

Nitrification is a key process in the nitrogen (N) cycle that converts ammonia (NH_3_) to nitrate (NO_3_^-^). The first and rate-limiting step is conducted by ammonia-oxidizing bacteria (AOB), archaea (AOA), and comammox (complete ammonia-oxidizing) bacteria via the ammonia monooxygenase (AMO)-dependent oxidation of NH_3_ (Hyman and Wood, 1983; Könneke et al., 2005; Tourna et al., 2011; Daims et al., 2015; van Kessel et al., 2015). The protonated form of NH_3_, ammonium (NH_4_^+^), is together with NO_3_^-^ the main form of N used by plants. However, in contrast to NH_4_^+^, NO_3_^-^ does not bind well to the negatively charged soil particles and leaches into deeper soil layers, where it is no longer available for plant uptake. As a result, an important part of the N-fertilizers applied in agriculture are lost, which leads to the eutrophication of groundwater, drinking water and recreational waters, or toxic algal blooms and biodiversity loss. Moreover, NO_3_^-^ can be transformed into nitric oxide (NO) and finally the very strong greenhouse gas nitrous oxide (N_2_O) during anaerobic denitrification (Galloway et al., 2003; Hu et al., 2015). Intermediate metabolites of NH_3_ oxidation can also be reduced to N_2_O via biotic or abiotic nitrifier denitrification (Wrage-Mönnig et al., 2018; Stein, 2019; Prosser et al., 2020). To prevent N loss, NH_4_^+^ is preferred over NO_3_^-^ in soil. As ammonia-oxidizing microorganisms compete with plants for NH_3_/NH_4_^+^, the use of nitrification inhibitors can enhance the nitrogen use efficiency (NUE) of plants. This increases crop yields, and of great importance as well, strongly reduce greenhouse gas emission from agriculture (Linquist et al., 2013; Abalos et al., 2014; Alonso-Ayuso et al., 2016; Sun et al., 2016; Yang et al., 2016).

It is generally assumed that NH_3_ oxidation in agricultural soils is mainly dependent on AOB. Despite a lower affinity, the oxidation rate of the bacterial AMO enzyme is in general higher than those of AOA or comammox bacteria, giving AOB an advantage in N-rich soils such as in agriculture (Beeckman et al., 2018). Nevertheless, a number of observations support possible importance of AOA as well. First, there is a lot of variety in the kinetic properties of the archaeal AMO enzyme, hence at least certain AOA might be responsible for nitrification in agricultural soils (Beeckman et al., 2018; Jung et al., 2022; Beeckman et al., 2023a). Secondly, AOA dominate certain agricultural soils (Duan et al., 2019; Huang et al., 2021). Finally, certain conditions, including treatment with 3,4-dimethylpyrazole phosphate (DMPP), a commonly used nitrification inhibitor, favor the growth of AOA (Hink et al., 2018; Fan et al., 2019; Yang et al., 2021; Yin et al., 2021; Fan et al., 2022) and will increase their contribution to nitrification. The latter makes sense, as the (AOB-targeting) nitrification inhibitor blocks the main competitors of the AOA, and makes that the substrate, otherwise used by AOB, is suddenly available for AOA. This also implies, however, that the use of nitrification inhibitors at the end promotes nitrification, at least by AOA, which are not targeted by DMPP.

Indeed, besides 2-chloro-6-trichloromethylpiridine (nitrapyrin), commonly used nitrification inhibitors, including dicyandiamide (DCD) and 3,4-dimethylpyrazole phosphate (DMPP) all target AOB, but not AOA (Lehtovirta-Morley et al., 2013; Shen et al., 2013). And for nitrapyrin it is actually not clear whether it effectively targets AOA: experiments in soil showed no effect of nitrapyrin on the AOA abundance in soil (Hayden et al., 2021), while co-application of nitrapyrin with an AOA inhibitor shows an additive effect (Beeckman et al., 2023b), indicating that nitrapyrin does not (completely) inhibit AOA in soil. Other AOA-targeting inhibitors cannot be used in the field due to their gaseous state, their aspecificity and/or their formation of reactive molecules (Taylor et al., 2015; Papadopoulou et al., 2020; Wright et al., 2020; Huang et al., 2021; Beeckman et al., 2023a). Hence, to improve nitrification inhibition and further reduce N pollution, there is a need for new types of nitrification inhibitors that are not only effective against AOB, but also against AOA. However, currently only few assays are described that would enable efficient testing of candidate AOA-targeting inhibitors (Schatteman et al., 2022; Beeckman et al., 2023b). As AOA are very sensitive to small perturbations, there are many caveats to consider. This is especially important when requiring high robustness, which is desired for screening purposes.

We recently screened approximately 45,000 small molecules to identify new AOA-targeting nitrification inhibitors, using both the AOA *Nitrosophaera viennensis* and an AOA-enriched community (Beeckman et al., 2023b). We here present the two screening assays. Our primary aim is to provide a detailed outline of the step-by-step methodologies to contribute to a good understanding of their practical implications. Specifically, we present the assays, elucidate possible pitfalls associated with their implementation and validate their utility in evaluating both established nitrification inhibitors and new variants of the recently discovered AOA-targeting nitrification inhibitor SIAS (4-[1,6-bis(propan-2-yl)-1H-pyrazolo[3,4-b]pyridine-4-carbonyl]thiomorpholine) (Beeckman et al., 2023b). The ultimate goal of this work is to facilitate the identification and testing of new nitrification inhibitors specifically targeting AOA, a group that has been historically undervalued. Such new types of inhibitors are important to increase nitrification inhibition efficiency, thereby promoting sustainability in agricultural contexts and plant production.

## 2 Materials and methods

### 2.1 Microbial cultures

*Nitrososphaera viennensis* EN76^T^ was kindly provided by Christa Schleper (University of Vienna) and ABIL (Ammonia-Binding Inoculum Liquid), an aqueous suspension containing a highly active, nitrifying microbial consortium (Grommen et al., 2002), was obtained from Avecom nv (Belgium).

### 2.2 Nitrite measurements

Nitrite was measured via a colorimetric Griess assay (Griess, 1879), by adding 15 µl modified Griess reagent (Sigma-Aldrich, Cat. No. G4410) to 15 µl sample in a flat-bottom 384-well plate (Cat. No. X7001, Low Profile Microplate, Molecular Devices). As the theoretical working range is between 0.43 and 64 μM, it is important to dilute the sample until it is in this working range and calculate the original concentration. For accurate quantitation of NO_2_^–^ levels in experimental samples, a Standard Reference Curve (SRC) was made with NaNO_2_ in the matrix or buffer of the experimental samples using an 8-point 1:2 dilution series starting from 100 μM (incl. a blank sample). After 15 minutes incubation in the dark at room temperature, absorbance (ABS) was measured at 540 nm using a spectrophotometer (EnVision, Perkin Elmer^®^).

### 2.3 Ammonium measurements

NH_4_^+^ was measured via a modified Berthelot’s reagent protocol. For this, 168 µl of (diluted) sample was first mixed with 35 µl of reagent A (0.5% (w/w) NaOH and 0.2% (v/v) NaClO) and then with 33 µl of reagent B (1% (w/w) salicylic acid, 0.5% (w/w) NaOH and 1.0236% (w/w) nitroprusside dihydrate) in a flat-bottom 96-well plates (Cat. No. 353075, Falcon^®^ 96-well Clear Flat Bottom Microplate, Corning^®^). Reagent A and B were stored in the dark at 4 °C and were maximally 3 weeks old. For accurate quantitation of NH_4_^+^, a 6-point 3:4 dilution series starting from 2.5 mM (NH_4_)_2_SO_4_ (incl. a blank sample) was prepared in triplicate as a SRC and subjected to the same treatment. After 30 minutes incubation, in the dark at room temperature, ABS was measured at 635 nm using a spectrophotometer (EnVision, Perkin Elmer^®^).

### 2.4 Cultivation and activation

*Nitrososphaera viennensis* EN76^T^ was grown in liquid batch cultures using Fresh Water Medium with 3 mM NH_4_^+^ (Tourna et al., 2011) in a light-sealed, 42 °C microbiological incubator without shaking. *N. viennensis* reached the late-log phase around 1 mM NO_2_^-^ (**Figure S1**). A 25-fold dilution factor was applied to measure the NO_2_^–^ level via the Griess assay. *N. viennensis* cultures were subcultivated at NO_2_^-^ concentration just above 1 mM (usually 7-day-old cultures) via a 1:400 dilution, ensuring relatively fast growth of the archaea without accumulation of possible toxic metabolites, and allowing continuous subcultivations. As *N. viennensis* is very sensitive to even minimal chemical contaminations, highly chemically resistant Quickstart universal containers STERILIN^®^ were used to propagate the cells in small batches and chemical-free Erlenmeyer flasks rinsed with 1% HCl to grow cultures in volumes up to 100 mL.

ABIL grows in flocs attached to CaCO_3_ particles in the medium (Grommen et al., 2002). It can be stored up to 6 months in 1 L plastic bottles at 5°C. To activate the ABIL inoculum, ABIL was sieved at least three times through an iron mesh (Ø 5 mm) and 600 mL was transferred to 1.5-L Erlenmeyer flasks where it was supplemented with 6 mL 250 mM (NH_4_)_2_SO_4_ to reach a final NH_4_^+^ concentration of 5 mM. The Erlenmeyer flasks were gently closed with a screwcap and sealed with Micropore tape (Micropore™ Surgical Tape 1530-1, 3M™) and incubated in the dark at 30 °C for 24 hours, while shaking (±160 rpm).

### 2.5 Statistics

Statistics were performed via Graphpad Prism and statistical tests are indicated in the figure legends.

To quantify the robustness and quality of the HTS assays, the Z-factor or Z-prime (Z’) was calculated. This is a measure to assess whether there is sufficient difference between the positive and negative controls. The Z’ usually varies between 0 and 1, but can be negative as well (see Equation 1). A Z’ of 1 is considered a perfect assay, but in practice should ideally be ≥ 0.5, which indicates a large dynamic range with small data variation. In other words, with a Z’ ≥ 0.5, the window between the positive and negative controls values is sufficiently large for screening, while an assay with a Z’< 0 shows overlap between positive and negative control values and cannot be used for screening (Zhang et al., 1999).


(Equation 1) $$Estimated\;Z'=1-\frac{3({\hat{\sigma }}_{p}+{\hat{\sigma }}_{n})}{\left|{\hat{\mu }}_{p}-{\hat{\mu }}_{n}\right|}\begin{array}{c} {\hat{\sigma }}_{p}\;=\text{standard deviation positive control}\\ \;{\hat{\sigma }}_{n}=\text{standard deviation negative control}\\ {\hat{\mu }}_{p}=\text{mean positive control}\\ {\hat{\mu }}_{n}=\text{mean negative control} \end{array}$$

### 2.6 Chemical structures

Images of chemical structures were generated via Jchem version 21.13.0.915 (ChemAxon – https://chemaxon.com/).

### 2.7 Step-by-step procedures high-throughput nitrification inhibition assays

#### 2.7.1 Nitrososphaera viennensis EN76^T^

1. Pre-cool a centrifuge that fits 50-mL centrifuge tubes at 5°C.

2. Distribute 50 mL late-log cultures in 50-mL conical centrifuge tubes.

**Note**: In our hands, it takes 7 days to reach the late log phase after subcultivation, which is characterized by a NO_2_^-^ level of about 1mM (**Figure S1**), but this should be verified first. Per final 96-well or 384-well assay plate, 100 mL of culture or two 50-mL Falcon tubes are required.

3. Centrifuge for 15 min at 4,000 rpm.

4. Carefully pour out all the supernatant of each centrifuge tube in a recipient without disturbing the cell pellet. If needed, small volumes of supernatant can be removed using a micropipette.

5. Add 10 mL Fresh Water Medium (FWM, **Supplementary Information**) to each centrifuge tube.

6. Close and shake the centrifuge tubes to dissolve the cell pellets.

7. Pool the content of all centrifuge tubes into a sterile glass bottle and close it.

8. Fill 96-well or 384-well plates with respectively 200 µL or 50 µL culture per well.

**Note**: We finally used 96-well plates in our assay to not exceed 0.25% DMSO and practical liquid handling (addition of 0.5 µl DMSO/compound). Distribution of culture should occur via gentle pipetting. We added 2 x 100 µl of culture using 150 µL wide-bore tips and slow pipetting speed on the liquid handling robot (**Supplementary Information**).

9. Add 0.5 µL or 0.125 µL of 99.95% DMSO (or other used solvent) to negative control wells of the 96-well or 384-well plate, respectively.

10. Add 0.5 µL or 0.125 µL of 40 mM 2-phenyl-4,4,5,5-tetramethylimidazoline-1-oxyl 3-oxide (PTIO) to reach a final concentration of 100 µM (or another positive control) to positive control wells of the 96-well or 384-well plate, respectively.

11. Add 0.5 µL or 0.125 µL of 1 mM compound to assay wells to reach a compound concentration of 2.5 µM.

**Note**: 2.5 µM resulted in our hands in a high hit rate, but other concentrations can be used by adapting the stock solutions (if compound solubility allows this) or the added volume. The latter will however affect the DMSO concentration and will affect the robustness of the assay (see below). For addition of 0.5 µL, we used a liquid handling robot equipped with a pin tool (**Supplementary Information**). In this case, in between additions, the pins are washed in sequence with 99.5% DMSO, Milli-Q water and 100% ethanol, and subsequently air-dried.

12. Seal all plates with surgical tape.

13. Stack the assay plates per 4 on top of a 96-well plate filled with 100 µL Milli-Q per well. Wrap with tinfoil and incubate in an incubator at 42°C for 24 h.

**Note**: There can be a huge position effect in the incubator. Add a water reservoir to the incubator to keep the humidity constant.

– 24 hours incubation period –

14. Fill 1 or 4 U-bottom 96-well plates (= intermediate plate(s)) with 144 µL FWM per well for each 96-well or 384-well assay plate, respectively.

15. Fill 1 transparent, flat bottom 384-well plate (= read-out plate) per 4 assay plates with 15 µL Griess reagent in each well.

16. Add 6 µL from each well of the assay plates in 144 µL FWM in the intermediate plates to obtain a 1:25 dilution.

17. Add 15 µL from each well in the intermediate plates to 15 µL Griess reagent in the read-out plates.

18. Incubate the read-out plates for 15 min in the dark at room temperature.

19. Measure the absorbance in the read-out plates at 540 nm.

20. Calculate relative nitrification:


Relative nitrification =ABS540(compound)−ABS540(positive control)ABS540(negative control)−ABS540(positive control)

#### 2.7.2 ABIL

1. Activate an ABIL culture by sieving ABIL inoculum three times using an iron mesh (Ø 5 mm), transferring to an Erlenmeyer flask, adding a final concentration of 2.5 mM (NH_4_)_2_SO_4_, gently closing the Erlenmeyer flask with a screwcap and surgical tape and incubate at 30°C for 24 hours, while shaking (±160 rpm).

**Note**: all NH_4_^+^ will be consumed after 24 hours, but the activation step ensures equal starting material and less intra-and interplate variability.

2. Stir the ABIL culture using a magnetic stirrer (speed: 600/min) to prevent the flocs from settling.

3. Fill all designated sample wells in a 96-well plate with 135 µL ABIL.

**Note**: We used an automatic dispenser equipped with a cassette with wide tubes to distribute the culture in the multi-well plates. In our experience, the use of a dispenser showed the best results for an equal distribution of the flocs and liquid over the different wells.

4. Add a SRC to the 96-well assay plates (see 2.3).

5. Add controls to the designated wells in the 96-well assay plates: 1.5 µL of 10 mM PTIO (positive control) and 1.5 µL of 99.95% DMSO (negative control).

6. Add 15 µL 25 mM (NH_4_)_2_SO_4_ to all wells (excl. SRC).

**Note**: To minimize intra-plate variability, we advise to add a relatively large volume of (NH_4_)_2_SO_4_ to the culture samples, e.g., 1:10 (v/v).

7. Add 1.5 µL compounds to all sample wells and 1.5 µL 99.95% DMSO to the SRC.

**Note**: It is important that the compounds are properly mixed with the flocs in the wells. If not, they do not distribute properly among the flocs in a well, as can be seen with colored compounds.

8. Seal all assay plates with surgical tape, stack per 4 and cover in tinfoil prior to incubation at 28°C for 24 h.

**Note**: Vigorous shaking is necessary to prevent sedimentation of the flocs and to assure oxygen saturation and complete NH_4_^+^ consumption over 24 h.

– 24 h incubation period –

9. Per assay plate, fill 1 transparent, flat bottom 96-well plate (= read-out plate) with 160 µL Milli-Q per well.

10. Dilute 8 µL culture sample from the assay plates in 160 µL in the read-out plates.

**Note**: It is crucial not to pipette any flocs from the assay plates to the read-out plates to prevent disturbance of the Berthelot reaction or read-out. The flocs must sediment in the plate and the pipette tip should be high enough to prevent flocs to be aspirated and low enough to prevent aspiration of air.

11. Add 35 µL Reagent A to the wells.

12. Add 33 µL Reagent B to the wells.

13. Incubate the read-out plates for 30 min in the dark at room temperature.

14. Measure the absorbance in the read-out plates at 635 nm.

15. Calculate relative nitrification:


Relative nitrification =ABS635(compound)−ABS635(positive control)ABS635(negative control)−ABS635(positive control)

## 3 Results

### 3.1 High-throughput nitrification inhibition assay using *Nitrososphaera viennensis* EN76^T^

#### 3.1.1 Procedure

We developed a high-throughput nitrification inhibition assay for *N. viennensis* that follows a methodology similar to previously described assays that use large volumes of cultures (e.g. Shen et al., 2013). However, our approach necessitated specific modifications to adapt the assay for miniaturization and robustness (see Materials and methods for details). In brief, our high-throughput assay starts from a late-log phase culture that is centrifuged and overconcentrated through the removal of supernatant and the addition of fresh medium. After resolving the pellet and possibly pooling multiple overconcentrated cultures, the culture is dispensed into a multi-well plate. Candidate nitrification inhibitors, negative controls with only the solvent and positive controls with, for example, the AOA-targeting PTIO are subsequently added to different wells. Following a 24-hour incubation period, a sample is pipetted from each well, appropriately diluted in an intermediate plate, and transferred to a read-out plate containing Griess reagent. After a 15-minute incubation, spectrophotometric measurements are conducted to quantify the formed NO_2_^-^ and as such the level of nitrification (inhibition). The effectiveness and robustness of the assay can be influenced by various parameters, such as the distribution method, solvent, and incubation conditions. Details on these factors, along with their potential impact on the assay, are provided in the Materials and methods section. It is recommended to conduct preliminary testing of these parameters to optimize the assay performance.

#### 3.1.2 DMSO effect and liquid handling

**Figure 1:**
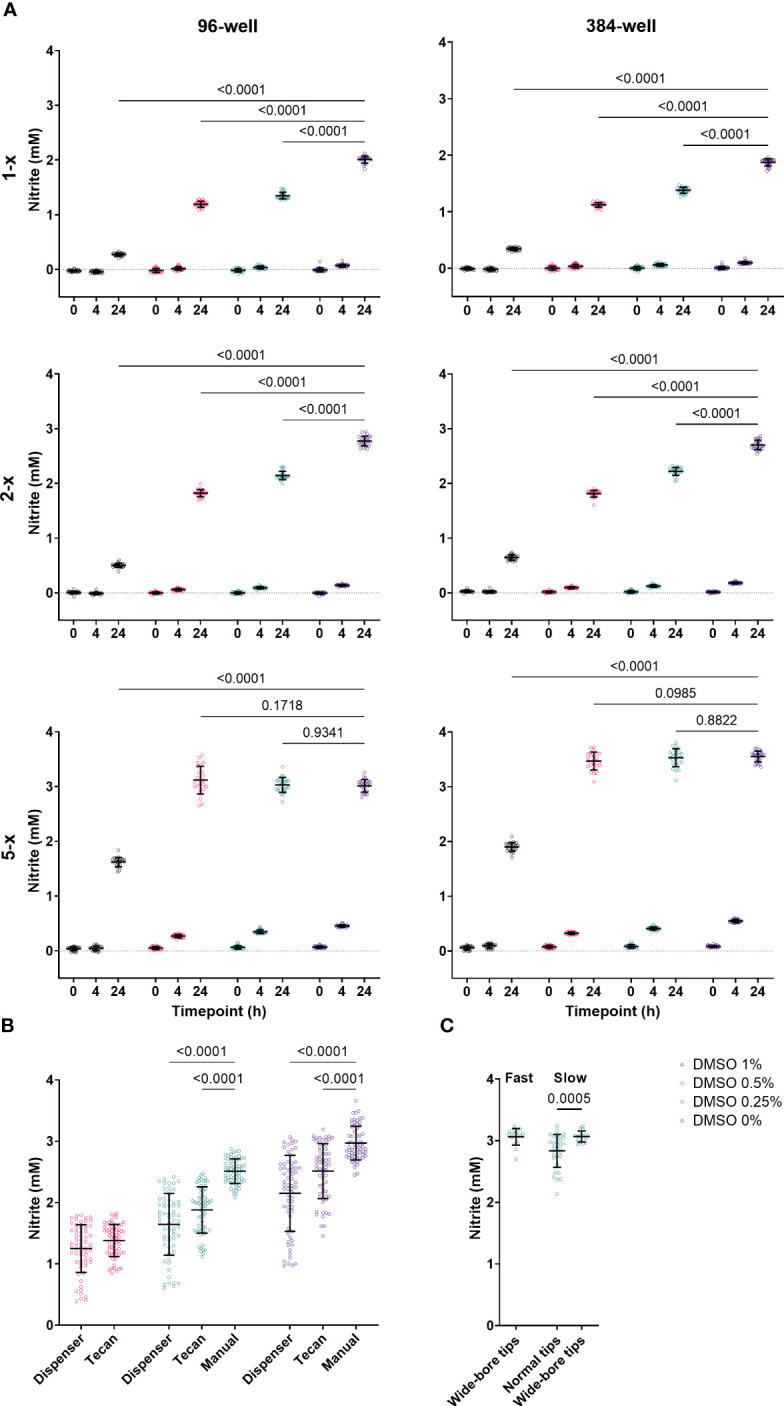
**(A)** Effect of 1%, 0.5%, 0.25% and 0% DMSO in 96- and 384-well plates at 0 h, 4 h and 24 h on 1-x, 2-x and 5-x overconcentrated *N. viennensis* cultures. *N. viennensis* was active in both 96- and 384-well plates. The growth inhibitory effect of DMSO on *N. viennensis* was positively correlated with the DMSO concentration in both 96- and 384-well plates. Only 5-x overconcentrated cultures reached the late-log or stationary phase within 24 h at DMSO concentrations of 0.5% or lower. Data (n = 24) were analyzed using a two-way ANOVA followed by a Dunnett test. P-values compared to the DMSO 0% condition at the 24 h measurements are presented. **(B)** Comparison of assay plate preparation via (gentle) manual pipetting, via a dispenser or via the standard settings on a liquid handling robot: automatic liquid handling has a significantly negative effect on NO_2_^-^ production at 24 h. Data (n = 64) were analyzed using a mixed-effects model followed by a Tukey test. P-values compared to the manual handling are presented. **(C)** Effect of pipetting speed and the use of wide-bore vs normal tips on NO_2_^-^ production at 24 h by *N. viennensis*. Variability can be reduced by using wide-bore tips and slow pipetting speed. Data for normal tips (n = 32) and for wide-bore tips (n = 16) was analyzed via a Mann-Whitney test and the p-value within the slow pipetting subset is represented. For all panels, open circles represent individual data points, lines represent the mean and error bars represent the standard deviation.

*N. viennensis* is known to be extremely sensitive to small perturbations, challenging the execution of a miniaturized nitrification inhibition assay. To illustrate this, we tested the effect of 1%, 0.5%, 0.25% and 0% DMSO, the compound solvent, in 96- and 384-well plates. Next to a 5-x overconcentrated culture as generally used (see Material and methods), we included a 1-x and 2-x overconcentrated culture as well. NO_2_^-^ levels were measured at 0 h, 4 h and 24 h after addition to the multi-well plates (**Figure 1A**). *N. viennensis* was active in both 96- and 384-well plates, though NO_2_^-^ production was slightly higher in the 384-well plates. Additionally, all DMSO concentrations had growth inhibitory effects in both 96- and 384-well plates, which was most clear at the 24 h measurement. In 5-x overconcentrated and hence highly active cultures, the cultures reached the late-log or stationary phase within 24 h at DMSO concentrations of 0.5% or lower. This maximum NO_2_^-^ production increases the possible difference between negative controls and nitrification inhibitors and thus the sensitivity of the assay. Still, lower DMSO concentrations result in less variation (**Figure 1A**), which is favorable for assay performance. Therefore, we opt to use a DMSO concentration of 0.25% (see Material and methods). Although *N. viennensis* was active in both 96- and 384-well plates, the latter might be less practical to use in high-throughput setting as it would require liquid handling of ≤ 0.25 µL or a pre-dilution step.

Next to DMSO, *N. viennensis* was also sensitive to the type of liquid handling, which is illustrated by comparing assay plate preparation via manual pipetting, via a dispenser or via the standard settings on a liquid handling robot. Manipulations with the dispenser or liquid handling robot had a strong negative effect on NO_2_^-^ production, which was even clearer if DMSO was added (**Figure 1B**). High-throughput assays require automation, and settings to make the liquid handling gentle seem to be essential in a *N. viennensis* assay. Indeed, reducing pipetting speed and the use of wide-bore tips had a clear positive effect on assay performance and reduced the variability (**Figure 1C**). As such, possibly any distribution handling that creates a high-rate flow is detrimental as it increases potential shearing of cells.

#### 3.1.3 Effects of known nitrification inhibitors

**Figure 2:**
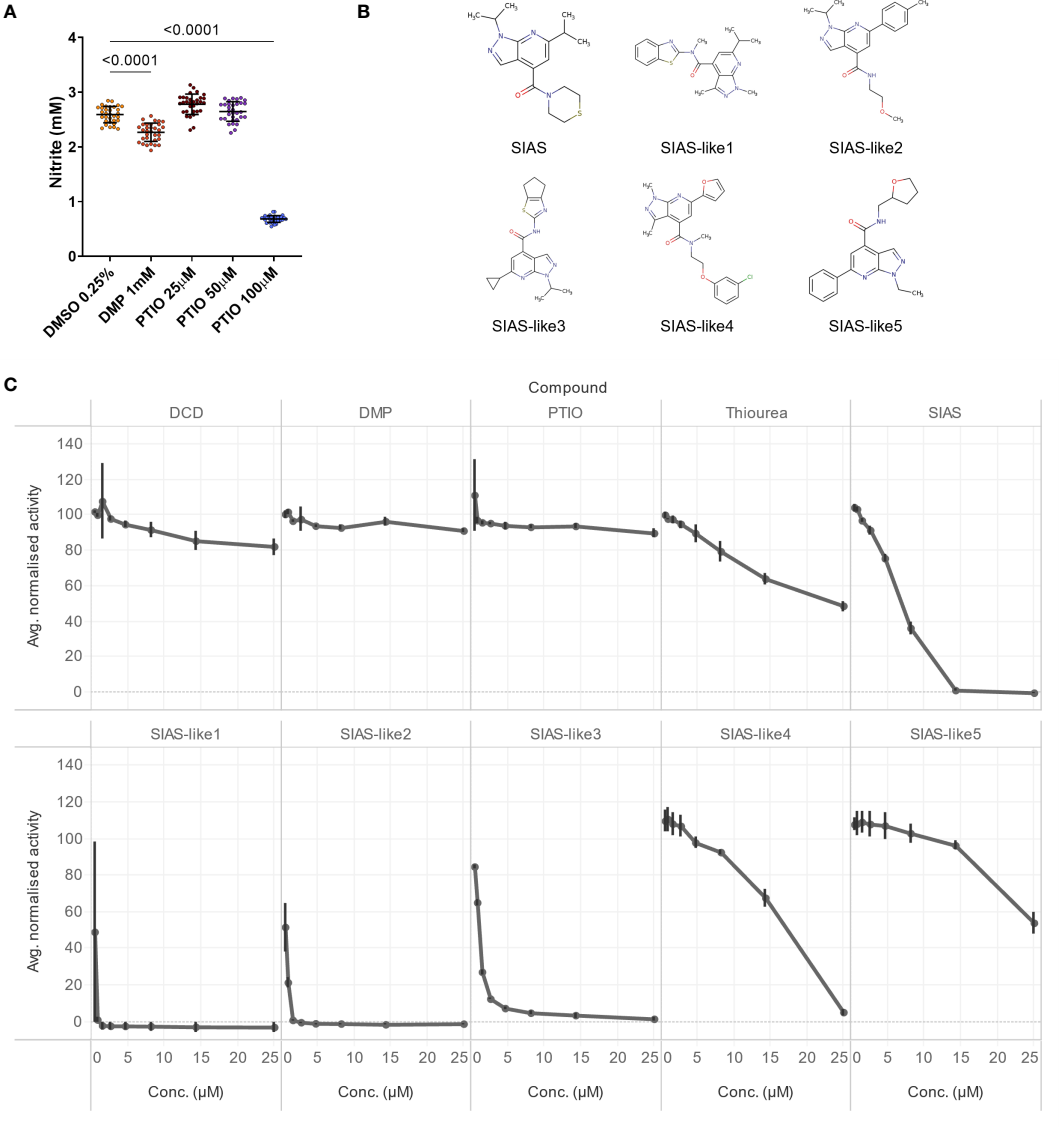
**(A)** NO_2_^-^ production in the *N. viennensis* assay upon treatment with DMP or PTIO. NO_2_^-^ production was strongly decreased by 100 µM PTIO but not by lower concentrations. A very high DMP (1 mM) concentration did result in a weak, though significant, inhibition. Data (n = 32) were analyzed using a one-way ANOVA followed by a Dunnett test. Circles represent individual datapoints, lines represent the mean and error bars represent the standard deviation. **(B)** Structural formulas of SIAS-like variants. **(C)** 8-point dose response inhibition test with known nitrification inhibitors DCD, DMP, PTIO and thiourea, compared to the new nitrification inhibitor SIAS and structural variants thereof. Tested concentrations were 25, 14, 8.2, 4.7, 2.7, 1.5, 0.87 and 0.50 µM. The line charts display the average normalized activity (n = 4), and error bars show the standard deviation. From the known inhibitors, only thiourea showed ± 50% nitrification inhibition at the highest tested concentration, while the new inhibitor SIAS and its structural variants 1 to 4 completely inhibited nitrification at 25 µM or lower.

Next, we tested the NO-scavenger PTIO (2-phenyl-4,4,5,5-tetramethylimidazoline-1-oxyl 3-oxide), the most frequently used archaeal nitrification inhibitor for *in vitro* experiments, in different doses, together with an extremely high dose of the AOB-targeting DMP (3,4-dimethyl pyrazole), the active substance of DMPP (**Figure 2A**). In contrast to 1 mM DMP, that showed marginal inhibition, 100 µM PTIO showed strong nitrification inhibition (**Figure 2A**). Lower concentrations of PTIO did not exhibit inhibition, however, the use of 100 µM PTIO as a positive control in the assay resulted in a substantial separation between positive and negative controls (**Figure 2A**), and we opted to use 100 µM PTIO as the reference to calculate the relative nitrification (see Material and methods).

To further leverage the assay, we evaluated several nitrification inhibitors in a concentration range from 0.5 µM to 25 µM (**Figures 2B, C**). As expected, DCD and DMP do not show clear inhibition. Also PTIO, at least at the tested concentrations did not show inhibition, while thiourea, described as a strong AOB inhibitor but weak AOA inhibitor (Beeckman et al., 2018), showed a weak inhibition at the higher concentrations. More importantly, the new archaeal nitrification inhibitor SIAS (Beeckman et al., 2023b) and several structural variants thereof showed highly effective inhibition (**Figures 2B, C**). SIAS-like 1 even completely inhibited nitrification in *N. viennensis* from 0.87 µM onwards (**Figures 2B, C**).

### 3.2 High-throughput nitrification inhibition assay using ABIL

#### 3.2.1 Procedure

To assess the impact of candidate nitrification inhibitors on a nitrifying community, we additionally developed a screening assay utilizing the ABIL (Ammonia-Binding Inoculum Liquid) inoculum. ABIL is generated by progressively enriching natural surface water with ammonium and nitrite, thereby selectively supporting the growth of nitrifying microorganisms (Grommen et al., 2002). While ABIL can be easily obtained commercially in large quantities, ensuring sufficient material for large screens, its practical implementation requires specific considerations (see **Materials and methods** for details). As a start, the microorganisms in ABIL grow in flocs, and to ensure an equal distribution of the flocs in the wells of a multi-well plate, a pre-sieved ABIL suspension should be continuously stirred during distribution, which preferentially occurs via a dispenser to minimize variation. Next, NH_4_^+^ and candidate nitrification inhibitors are added to the wells and incubated for 24 h. During this incubation, plates are subjected to shaking to avoid sedimentation of the flocs. As ABIL contains both ammonia- and nitrite-oxidizing microorganisms, NO_2_^-^ measurements cannot be used to assess the ammonia oxidation or the action of nitrification inhibitors. Therefore, we measured NH_4_^+^ via a modified Berthelot reaction, requiring the addition of two reagents to a diluted sample. Of important note, during the dilution step, where samples are taken from the assay plate, no flocs should be transferred as these interfere with the spectrophotometric read-out of the Berthelot reaction. Therefore, when using a liquid handling robot, the pipette tip height should be adjusted to prevent aspiration of flocs, and pipetting should occur after sedimentation of the flocs. Based on the NH_4_^+^ levels, the relative nitrification can be calculated. Similar as for the *N. viennensis* assay, the robustness of the assay can be influenced by various parameters, as presented in the Materials and methods section. It is therefore recommended to conduct a preliminary test before effectively applying the assay.

#### 3.2.2 Effect of known nitrification inhibitors

**Figure 3:**
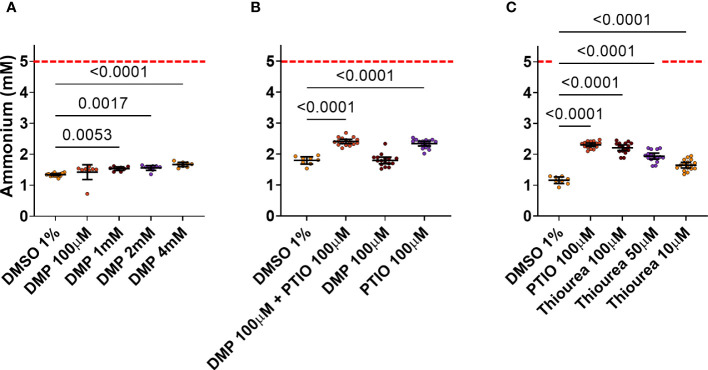
Effect of known nitrification inhibitors on NH_4_^+^ consumption by ABIL at 24 h after treatment. Dashed red line shows the theoretical NH_4_^+^ concentration at the start of the experiment. **(A)** 1, 2 and 4 mM DMP significantly inhibit nitrification in ABIL, but the window between these inhibitors (n = 8) and DMSO 1% (n = 16) is not sufficiently large to be used as a positive control in HTS. **(B)** 100 μM PTIO significantly inhibit nitrification in ABIL, but this was not enhanced when co-applied with 100 μM DMP (n = 16 for inhibitors, n = 8 for DMSO 1%). **(C)** 100 μM thiourea and 100 μM PTIO are equally strong inhibitors of nitrification in ABIL (n = 16 for inhibitors, n = 8 for DMSO 1%). All data were analyzed using a one-way ANOVA followed by a Dunnett test. Significant p-values compared to the DMSO control are shown. Circles represent individual datapoints, lines represent the mean and error bars represent the standard deviation.

Four different concentrations of DMP were tested as possible positive controls. DMP is known to strongly inhibit AOB nitrification at 100 µM or less (Beeckman et al., 2023b), but only very high concentrations (4, 2 and 1 mM) of DMP showed a significant reduction in the NH_4_^+^ consumption by ABIL (**Figure 3A**). None of them showed a large difference with the negative control (DMSO 1%), while theoretically, strong inhibition should result in a NH_4_^+^ level of 5 mM. Hence, it seems that the strong nitrification inhibitor DMP is not able to efficiently inhibit nitrification in ABIL, and cannot be used as a positive control for high-throughput screening (HTS) (**Figure 3A**). qPCR analysis targeting bacterial and archaeal *amoA* showed a clear dominance of archaeal *amoA* compared to bacterial *amoA* (**Figure S2**, **Supplementary Information**), illustrating that the community is strongly dominated by AOA, and probably explaining why it is not sensitive to DMP. The archaeal inhibitor PTIO showed indeed stronger inhibition, but a lot of the supplied NH_4_^+^ is still consumed (**Figures 3B, C**). To target both AOB and AOA in ABIL, we tested the archaeal inhibitor PTIO in combination with DMP. The co-application had the same effect as single application of PTIO (**Figure 3B**). Even a concentration up to 400 μM of PTIO did not further increase inhibition (data not shown), illustrating that 100 μM resulted in a maximum inhibitory effect. Likewise, the strong nitrification inhibitor thiourea did not surpass PTIO (**Figure 3C**). Hence, no tested nitrification inhibitor, nor combinations, showed a complete nitrification inhibition on this nitrifying community.

### 3.3 High-throughput compound screening

**Figure 4:**
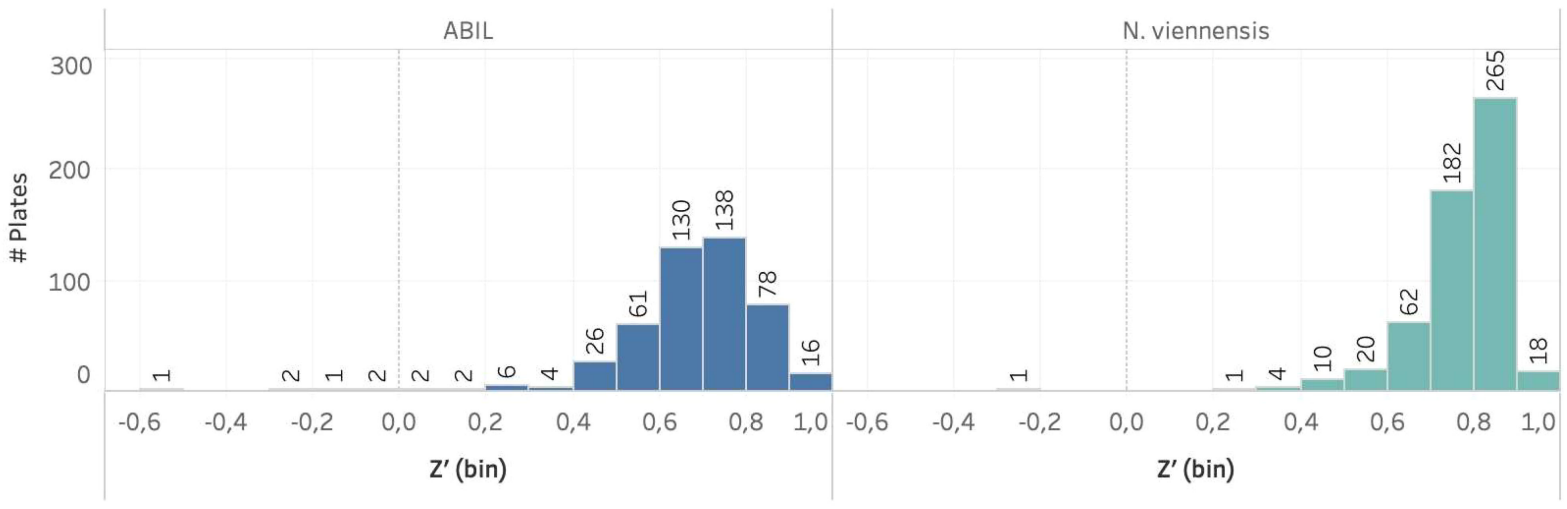
Bar chart showing the number of primary screen assay plates per model system with their respective Z’-values. In both model systems, a large majority of the plates had a Z’ value ≥ 0.5. It should be noted that several assay plates with Z’-values between 0 and 0.5 are common during such large screening campaigns and are thus also acceptable.

The two assays were challenging to develop, as illustrated by the need for specific adaptations to address difficulties, such as the impact of DMSO in the case of *N. viennensis*, or the presence of flocs when using ABIL. Nevertheless, the specific adjustments made in the final procedures, including the use of highly concentrated *N. viennensis* cultures, or the specific liquid handling settings to avoid flocculation issues (see Material and methods), showed high robustness and were suitable to implement in a large high-throughput screen. To support this, we calculated Z’-values - a measure to evaluate the separation between the signal from positive controls and the noise from negative controls in an assay (Zhang et al., 1999) - of approximately 500 multi-well plates used within a HTS campaign (Beeckman et al., 2023b). The large majority of assay plates within the two screening assays had Z’-values ≥ 0.5 (**Figure 4**), validating the methodology. Especially for the *N. viennensis* assay, the Z’-values were often close to 1, suggesting a near perfect assay. Even the ABIL assay, for which we had issues to find good positive controls, showed Z’-values primarily between 0.6 and 0.8, validating the assay’s reliability to discriminate between active and inactive compounds.

## 4 Discussion

We developed two new and unique miniaturized nitrification inhibition assays suitable for HTS to identify AOA-targeting nitrification inhibitors. We have presented their procedures and validated their suitability to evaluate nitrification inhibitors. New nitrification inhibitors are usually found by testing compounds on soil or AOB, but not on AOA or nitrifying communities. As a result, nitrification inhibitors that are applied in agriculture all target AOB. Although AOB typically dominate agricultural soils, AOA can surpass AOB or gain a competitive advantage when current (AOB-targeting) nitrification inhibitors are applied (Leininger et al., 2006; Prosser and Nicol, 2008; Lehtovirta-Morley, 2018; Gao et al., 2020; Zhao et al., 2020; Huang et al., 2021; Beeckman et al., 2023a). Hence, there is a need to identify new nitrification inhibitors that target AOA or that have broad target range and are effective across diverse microbial contexts. Our nitrification inhibition assays, utilizing the AOA *N. viennensis* or a nitrifying community like ABIL, could facilitate the discovery of such inhibitors.

We showed different challenges in applying HTS assays using AOA and experienced that *N. viennensis* is very sensitive to small perturbations. As such, the compound solvent DMSO strongly affects *N. viennensis* and may confound robustness of the assays. Increased cell densities can be used to limit the confounding effect. Conversely, gentle liquid handling is important: a high variability and lower nitrification rate was observed when the assay plates were filled by a liquid handling robot at standard pipetting speed using standard tips. Most probably, these settings caused shearing of cells, leading to reduced NO_2_^-^ production. Therefore, slow pipetting and wide-bore tips were used in the final assay.

A limited set of nitrification inhibitors was previously tested on AOA, and usually this showed that AOA are not susceptible to DCD and DMP (Lehtovirta-Morley et al., 2013; Shen et al., 2013), which was confirmed in our assays. The important role of NO as an electron donor in the archaeal NH_3_ oxidation pathway possibly explains its sensitivity to PTIO, an NO-scavenger (Martens-Habbena et al., 2015; Sauder and Neufeld, 2016). In our assay, 100 µM PTIO fully inhibited nitrification in *N. viennensis*. Of interest, SIAS and especially the variants SIAS-like1 to 3 show highly effective inhibition of *N. viennensis*, and could be useful controls in future applications of our assay.

Assay development in ABIL was largely complicated by the fact that it grows in flocs attached to the surplus CaCO_3_ particles in the medium (Grommen et al., 2002). To assure equal nitrification activity in all wells, these flocs had to be distributed equally over all wells in the 96-well plates. In our hands, this was only achieved by using a dispenser and rigorously shaking the bottle containing ABIL while dispersion was in operation. Prior to this, ABIL had to be sieved to prevent large flocs from clogging the tubing. Moreover, the flocs disturbed the NH_4_^+^ read-out via the Berthelot assay. Possibly, they did not fully sediment in the assay plates and as such disrupted the read-out either by blocking the pipet tips, by sedimentation in the read-out plates or via scattering of the transmitted light. Adding a larger volume of the ammonium sulphate solution reduced intra-plate read-out variability, as this possibly aided easier diffusion of NH_4_^+^ within the flocs, while also slightly reducing the percentage of flocs in each well. At the end, these adjustments showed to be valuable as we were able to validate the robustness of the assay. Indeed, the Z’-values primarily ranging from 0.6 to 0.8 indicate an excellent assay (Zhang et al., 1999). Z’-values are typically used to assess the assay quality for HTS, for example in drug discovery screens, where high reproducibility of the assay performance is required (Iversen et al., 2006). A Z’ > 0.5 shows a large separation band between the positive and negative control, showing that the assay can be reliably used to assess nitrification inhibitors.

In 2002, prior to the discovery of AOA, the major active nitrifying organisms in the ABIL consortium were said to be *Nitrosococcus*, *Nitrosomonas*, and *Nitrobacter* species based on fluorescence *in situ* hybridization (Grommen et al., 2002; Könneke et al., 2005). ABIL, however, seemed to be more sensitive to PTIO than to DMP or thiourea, which pointed to a possible dominance of AOA, at least in the batch used here. This was also confirmed via a qPCR analysis targeting *amoA*. In contrast to *N. viennensis*, even PTIO was not able to fully inhibit nitrification by this AOA-dominated community, indicating that indeed new and better types of nitrification inhibitors are necessary to further enhance nitrification inhibition in complex nitrifying communities. As a result, PTIO could not be used as a complete positive control, but it was used as a reference compound to evaluate the nitrifying activity of the culture in each assay plate.

The assay development resulted in detailed procedures for two miniaturized nitrification inhibition assays. To our knowledge, this has never been done for AOA or nitrifying communities before, at least not with proven effectiveness and quality that make the assays suitable for large-scale HTS, as supported by the Z’-values. Furthermore, the assay was here successfully used to quantify the effect of the new archaeal nitrification inhibitor SIAS and several structural variants in dose-response on *N. viennensis*. The different points-of-attention discussed in the assay development, especially regarding the use of known nitrification inhibitors as adequate positive controls, as well as the detailed high-throughput procedures form a guideline and basis to develop new, similar screening assays on elusive microorganisms. This approach holds promise in the identification of new and novel Nutrient-Use-Enhancing molecules, and may contribute significantly to the development of eco-friendly and efficient strategies for managing nitrification and promoting nutrient utilization of crops in agricultural systems.

## 5 Conclusion

We have introduced and detailed the development of two miniaturized nitrification inhibition assays designed for HTS or testing of small molecules. Traditionally, the search for new nitrification inhibitors has focused on testing few compounds on soil or AOB, and not AOA or complex nitrifying communities. Our assays address this gap, offering advantages in identifying inhibitors targeting different nitrifier groups to increase nitrification inhibition efficiency effective in complex microbial contexts.

These innovative assays were successfully applied to identify new nitrification inhibitors (Beeckman et al., 2023b) and quantify the effects of structural variants of the archaeal nitrification inhibitor SIAS on *N. viennensis*, demonstrating their effectiveness in large-scale HTS campaigns.

## Data availability statement

The original contributions presented in the study are included in the article/**Supplementary Material**. Further inquiries can be directed to the corresponding authors.

## Author contributions

FB: Conceptualization, Formal analysis, Investigation, Methodology, Writing – original draft, Writing – review & editing. AD: Conceptualization, Formal analysis, Investigation, Methodology, Writing – review & editing. ADK: Methodology, Writing – review & editing. DA: Conceptualization, Supervision, Writing – review & editing. TB: Conceptualization, Supervision, Writing – review & editing. HM: Conceptualization, Investigation, Supervision, Writing – original draft, Writing – review & editing.

## Supplementary material

Supplementary Figure 1 NO_2_^-^ production by N. viennensis growth from lag-phase until late-log phase in 7 days and in stationary phase at 10 days (n = 5). Error bars represent standard error.

Supplementary Figure 2 Abundances of the different ammonia-oxidizing functional groups in ABIL. The bar plot shows the average amoA copy numbers of AOB (green), AOA (yellow), and clade A (dark red) and clade B (light red) comammox bacteria in ABIL.
