# Supplementary material for: High-throughput assays to identify archaea-targeting nitrification inhibitors
Source: Front Plant Sci. 2024 Jan 8;14:1283047. doi: 10.3389/fpls.2023.1283047 (PMC10800436; doi:10.3389/fpls.2023.1283047)
Supplement: Supplementary file 1 [file DataSheet_1.docx]

**High-throughput assays to identify archaea-targeting nitrification inhibitors**

Fabian Beeckman^1,2^, Andrzej Drozdzecki^3,4^, Alexa De Knijf^1,2^, Dominique Audenaert^3,4^, Tom Beeckman^1,2,*^, Hans Motte^1,2,*^

^1^Ghent University, Department of Plant Biotechnology and Bioinformatics, Technologiepark 71, 9052 Ghent, Belgium

^2^VIB Center for Plant Systems Biology, Technologiepark 71, 9052 Ghent, Belgium

^3^VIB Screening Core, Technologiepark 71, 9052 Ghent, Belgium

^4^Ghent University Centre for Bioassay Development and Screening (C-BIOS), 9052 Ghent, Belgium

*Corresponding authors: [tom.beeckman@psb.ugent.be](mailto:tom.beeckman@psb.ugent.be), [hans.motte@psb.ugent.be](mailto:hans.motte@psb.ugent.be)

**Supplementary Information**

1. **qPCR**

Soil samples (300 mg) or ABIL samples (300 mg pellet) were used for DNA extraction with the DNeasy PowerSoil Pro kit (Qiagen). Extracted DNA concentration and quality were determined by spectrophotometry (NanoDrop™ One, Thermo Scientific). Each qPCR reaction had a total volume of 5 µL and contained 0.5 µL DNA (50 ng/µL), 2 µL primer mix (0.5 µM, except for comammox primers 2 µM) of the forward and reverse primer and 2.5 µL 2xSYBR Green Mix. Used primers sets are listed in Table S1. qPCR reactions were run using a LightCycler480 (Roche). Relative quantities were calculated by means of standard curves prepared from serial dilutions of 10^7^ to 10^3^ gene copies μL^−1^ of linearized plasmids with insertions of the target genes, and the copy number of target genes per gram of ABIL pellet was calculated as described previously (Behrens et al., 2008).

1. **Fresh Water Medium (FWM) Growth medium**

**MATERIALS**

**EQUIPMENT**

- 7 x 1 L Schott DURAN borosilicate glass bottles
- 4 x 100 mL Schott DURAN borosilicate glass bottles
- spoons
- weighing dishes
- balance
- micropipette(s)
- micropipette tips
- Pipetboy
- pipette(s)
- 2 x 50 mL syringes
- 10 x 15 mL Falcon tubes
- pH meter
- stirrer + heating
- autoclave
- 500 mL bottle top filter (VWR Filter Upper Cup, European Article No. 514-0340)

**REAGENTS**

- Milli-Q water
- NaCl
- MgCl_2_ 6H_2_O
- CaCl_2_ 2H_2_O
- KH_2_PO_4_
- KCl
- HCl
- H_3_BO_3_
- MnCl_2_ 4H_2_O
- CoCl_2_ 6H_2_O
- NiCl_2_ 6H_2_O
- CuCl_2_ 2H_2_O
- ZnSO_4_ 7H_2_O
- Na_2_MoO_4_ 2H_2_O
- FeNaEDTA
- Biotin
- Folic acid
- Pyridoxine HCl
- Thiamine HCl
- Riboflavin
- Nicotinic acid
- Pantothenic acid
- P Aminobenzoic acid
- Choline Chloride
- Vitamin B12
- KOH
- NaHCO_3_
- Carbenicillin
- Kanamycin
- NH_4_Cl
- HEPES
- Na-pyruvate

**PROCEDURE**

To minimize contaminations, clean all necessary glassware and stirring-bars carefully by rinsing with 1 % HCl (diluted in Milli-Q water) and 3 times rinsing with Milli-Q water afterwards.

**BASIC FWM**

Important: BASIC FWM solution should be free of any precipitations.

1. Put 1 L Milli-Q water in a glass Schott bottle.
2. Add:

1.0 g NaCl

0.4 g MgCl_2_ 6H_2_O

0.1 g CaCl_2_ 2H_2_O

0.2 g KH_2_PO_4_

0.5 g KCl

1. Autoclave the BASIC FWM and cool to room temperature.

**FWM**

1. Aseptically add in sequence per 1 L of BASIC FWM:

1.0 mL Modified Trace Elements

1.0 mL FeNaEDTA Solution

1.0 mL Vitamin Solution

2.0 mL Sodium Bicarbonate NaHCO_3_ (1 M)

2.0 mL Kanamycin (100 mg/mL)

2.0 mL Carbenecillin (100mg/mL)

3.0 mL NH_4_Cl (1 M)

10 mL HEPES Buffer (1 M)

1.0 mL Na-Pyruvate (0.1 M)

1. The pH of the medium should be ~7.5.

**MODIFIED TRACE ELEMENTS**

Important: this solution contains no iron! Store dark at 4^o^C. Stable for ± 6 months.

1. Put 987 mL Milli-Q water in a glass Schott bottle.
2. Add:

8 mL HCl (~ 12.5M)

30 mg H_3_BO_3_

100 mg MnCl_2_ 4H_2_O

190 mg CoCl_2_ 6H_2_O

24 mg NiCl_2_ 6H_2_O

2 mg CuCl_2_ 2H_2_O

144 mg ZnSO_4_ 7H_2_O

36 mg Na_2_MoO_4_ 2H_2_O

1. Autoclave.

**FeNaEDTA SOLUTION (7.5 mM)**

Store dark at 4^o^C. Stable for ± 6 months.

1. Put 1 L Milli-Q water in a glass Schott bottle.
2. Add 2.753 g FeNaEDTA.
3. Sterilize by filtration.

**VITAMIN SOLUTION**

Store dark at 4^o^C. Stable for ± 6 months.

1. Put 1 L Milli-Q water in a glass Schott bottle.
2. Add:

0.02 g Biotin

0.02 g Folic acid

0.10 g Pyridoxine HCl

0.05 g Thiamine HCl

0.05 g Riboflavin

0.05 g Nicotinic acid

0.05 g DL Pantothenic acid

0.05 g P Aminobenzoic acid

2.00 g Choline Chloride

0.01 g Vitamin B12

1. Adjust to pH 7 with KOH.
2. Sterilize by filtration.

**SODIUM BICARBONATE (1 M)**

Store dark at 4^o^C. Stable for ± 6 months.

1. Put 1 L Milli-Q water in a glass Schott bottle.
2. Add 8.4 g NaHCO_3_.
3. Heat the solution a bit to easily dissolve the powder.
4. Autoclave.

**CARBENECILLIN (100 mg / mL)**

1. Put 50 mL Milli-Q water in a 100 mL glass Schott bottle.
2. Add 5 g Carbenicillin.
3. Suck up the solution with a 50 mL syringe.
4. Put a filter on the syringe.
5. Pout in 5 x 15 mL Falcon tubes.

**KANAMYCIN (100 mg / mL)**

1. Put 50 mL Milli-Q water in a 100 mL glass Schott bottle.
2. Add 5 g Kanamycin.
3. Suck up the solution with a 50 mL syringe.
4. Put a filter on the syringe.
5. Pout in 5 x 15 mL Falcon tubes.

**NH_4_Cl (1 M)**

1. Put 50 mL Milli-Q water in a glass Schott bottle.
2. Add 2.6745 g NH_4_Cl.
3. Sterilize by filtration.

**HEPES BUFFER**

If 1 mL of this solution is added to 100 mL of FWM (containing all additives besides HEPES) the pH should turn out ~7.6 at 30 ^°^C. Otherwise, adjust the pH of the HEPES solution as necessary with 10 M NaOH or concentrated HCl. Store in the dark at 4 °C.

1. Put ~ 300 mL Milli-Q water in a glass Schott bottle.
2. Slowly add 119,2 g HEPES until completely dissolved while stirring vigorously.
3. Fill up to 500 mL with Milli-Q water.
4. Sterilize by filtration.

**SODIUMPYRUVATE (100 mM)**

1. Put 100 mL Milli-Q water in a glass Schott bottle.
2. Add 1.1 g Na-pyruvate.
3. Sterilize by filtration.
   1. **Materials high-throughput nitrification inhibition assays**

| **Material** | **Product details** |
| --- | --- |
| Liquid handling robot | Freedom EVO® (Tecan) |
| U-bottom 96-well plates | Cat. No. 353077, Falcon® 96-well Clear Round Bottom Microplate, Corning® |
| Flat bottom 384-well plate | Cat. No. X7001, Low Profile Microplate, Molecular Devices |

**References**

Behrens, S., F., A. M., J., M. P., Andrew, S., E., D. M., Lew, S., et al. (2008). Monitoring abundance and expression of “*Dehalococcoides*” species chloroethene-reductive dehalogenases in a tetrachloroethene-dechlorinating flow column. *Appl. Environ. Microbiol.* 74, 5695–5703. doi: 10.1128/AEM.00926-08.
