# Supplementary figures and images for: High-throughput assays to identify archaea-targeting nitrification inhibitors
Source: Front Plant Sci. 2024 Jan 8;14:1283047. doi: 10.3389/fpls.2023.1283047 (PMC10800436; doi:10.3389/fpls.2023.1283047)

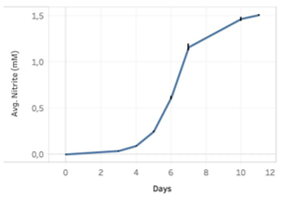

Supplement: Supplementary Figure 1 — NO2 - production by N. viennensis growth from lag-phase until late-log phase in 7 days and in stationary phase at 10 days (n = 5). Error bars represent standard error. [file Image_1.tif]

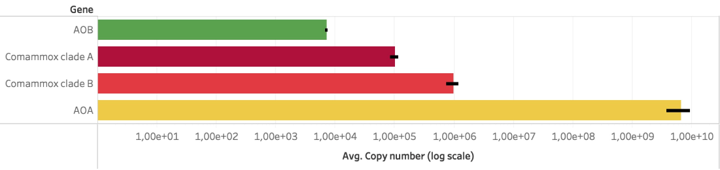

Supplement: Supplementary Figure 2 — Abundances of the different ammonia-oxidizing functional groups in ABIL. The bar plot shows the average amoA copy numbers of AOB (green), AOA (yellow), and clade A (dark red) and clade B (light red) comammox bacteria in ABIL. [file Image_2.tif]
